# Bioaerosols from a Food Waste Composting Plant Affect Human Airway Epithelial Cell Remodeling Genes

**DOI:** 10.3390/ijerph110100337

**Published:** 2013-12-24

**Authors:** Ming-Wei Chang, Chung-Ru Lee, Hsueh-Fen Hung, Kuo-Sheng Teng, Hsin Huang, Chun-Yu Chuang

**Affiliations:** 1 Department of Biomedical Engineering and Environmental Sciences, National Tsing Hua University, No. 101, Sec. 2, Kuang-Fu Rd., Hsinchu City 30013, Taiwan; E-Mails: awei680420@gmail.com (M.-W.C.); g9612512@oz.nthu.edu.tw (C.-R.L.); hsinh914@gmail.com (H.-H.); 2 Department of Environmental Engineering and Health, Yuanpei University, No. 306, Yuanpei St., Xiangshan Dist., Hsinchu City 30015, Taiwan; E-Mails: vivianhung@mail.ypu.edu.tw (H.-F.H.); a8959020@hotmail.com (K.-S.T.)

**Keywords:** bioaerosol, endotoxin, *Aspergillus fumigatus*, airway remodeling, gene expression, composting plant, particulate matter

## Abstract

The composting procedure in food waste plants generates airborne bioaerosols that have the potential to damage human airway epithelial cells. Persistent inflammation and repair responses induce airway remodeling and damage to the respiratory system. This study elucidated the expression changes of airway remodeling genes in human lung mucoepidermoid NCI-H292 cells exposed to bioaerosols from a composting plant. Different types of microorganisms were detectable in the composting plant, using the agar culture method. Real-time polymerase chain reaction was used to quantify the level of *Aspergillus fumigatus* and the profile of remodeling genes. The real-time PCR results indicated that the amount of *A*. *fumigatus* in the composting hall was less than 10^2^ conidia. The endotoxins in the field bioaerosols were determined using a limulus amebocyte lysate test. The endotoxin levels depended on the type of particulate matter (PM), with coarse particles (2.5–10 μm) having higher endotoxin levels than did fine particles (0.5–2.5 μm). After exposure to the conditioned medium of field bioaerosol samples, NCI-H292 cells showed increased pro-inflammatory interleukin (IL)-6 release and activated epidermal growth factor receptor (EGFR), transforming growth factor (TGF)-β1 and cyclin-dependent kinase inhibitor 1 (p21^WAF1/CIP1^) gene expression, but not of matrix metallopeptidase (MMP)-9. Airborne endotoxin levels were higher inside the composting hall than they were in other areas, and they were associated with PM. This suggested that airborne bioaerosols in the composting plant contained endotoxins and microorganisms besides *A*. *fumigatus* that cause the inflammatory cytokine secretion and augment the expression of remodeling genes in NCI-H292 cells. It is thus necessary to monitor potentially hazardous materials from bioaerosols in food composting plants, which could affect the health of workers.

## 1. Introduction

Bioaerosols are a potential source of airborne biological agents associated with a wide range of public health problems [1]. Exposure to bioaerosol components (fungi, bacteria, mycotoxins, and endotoxins) in the working environment has emerged as a dominant health concern in some occupational settings such as wastewater treatment and composting facilities. Composting is a natural biological process to biodegrade organic waste such as food waste, paper, manure, and crop residues. Waste composting, an effective waste management processes, not only reduces the amount of waste going to landfills, but can also convert waste into a valuable soil amendments and improve the texture and fertility of soil [2]. Food waste composting is a process that collects food waste and mixes green waste to assist the natural degradation of these materials by microbial action [3]. However, there are concerns regarding the potential impact on health caused by airborne bioaerosol components such as endotoxins, bacteria (mesophiles and thermophiles), and fungi during the composting process (piling up, agitation, and fermentation). Once inside the terminal airways, bioaerosols can attach to epithelial cells in the airway and cause harmful effects. The U.S. National Institute for Occupational Safety and Health (NIOSH) has indicated that greenhouse workers are one of the two top priority groups affected by both upper and lower respiratory tract problems [4]. Symptoms of infection from microorganisms most often encountered in some work environments are mucous membrane irritation, allergic rhinitis and asthma, allergic alveolitis or organic dust syndrome [5,6]. Flannigan *et al*. [7] found that people with prolonged exposure to home bioaerosols typically acquired allergic lung diseases. A cohort study reported that workers exposed to organic dust from composting plants had a higher prevalence of mucosal membrane irritation of the eyes and upper airways [8]. Muller *et al*. [9] reported that short-term exposure of healthy young patients to organic dust in composting plants had a mild but measurable effect (increased blood neutrophilia) in eliciting an acute systemic alteration. In addition, the increase in allergic reactions was positively correlated to the composition of the dust and the duration of exposure [10,11,12,13,14]. 

Endotoxins are biologically active lipopolysaccharides (LPS), which are major constituents of the outer cell wall of Gram-negative bacteria, and represent critical components of bioaerosols [15,16]. Exposure to endotoxins (or their purified derivative LPS) at elevated concentrations causes occupational pulmonary diseases such as acute airway obstruction, hypersensitivity pneumonitis, and chronic bronchitis [1]. Inhalation of endotoxins in various workplaces (e.g., recycling factories, food processing factories, and composting plants) has been reported to cause fever, coughing, wheezing, headaches, nose and throat irritation, acute airway restriction, and inflammation [1,10,17,18]. Chronic exposure to endotoxins influences the development of non-atopic chronic obstructive pulmonary disease (COPD) and the severity of asthma [19]. Furthermore, endotoxins may be causal agents in various serious diseases such as sepsis and septic shock [20]. Among the various microorganisms found in bioaerosols from composting plant, fungi easily become opportunistic human pathogens, particularly *Aspergillus* [21]. Aspergillus is a group of molds, of which about 200 species have been identified. *A*. *fumigatus* is one of the most ubiquitous airborne saprophytic fungi [22]. As we know, *A*. *fumigatus* causes infection in humans more often than any other *Aspergillus* species (e.g., *A*. *Niger* and *A*. *Flavus*). The cell walls of both living and dead spores of *Aspergillus fumigatus* (*A*. *fumigatus*) contain mycotoxins or β-(1-3)-glucans [23,24]. *A*. *fumigatus* does not cause diseases unless the host has a compromised immune system, in which case it can cause allergic illnesses and severe life-threatening infections [25,26]. 

The respiratory tract epithelium provides a protective barrier against the external environment. Airway remodeling occurs when the airway epithelial cells undergo chronic inflammation, impaired epithelial barrier function and inadequate repair of damaged epithelia [27,28]. Airway remodeling is activated by cytokines, and acts through tissue repair or remodeling gene expression resulting from chronic or short-term exposure to inflammatory stimuli [29]. Activated airway epithelial cells are a source of hematopoietic cytokines, pro-inflammation cytokines and chemokines that generate chemotactic signals [30]. Matrix metallopeptidase (MMP)-9 is known to be involved in structural changes of the bronchial epithelium in response to a prolonged period of epithelial repair [31].In addition, transforming growth factor (TGF)-β and epidermal growth factor receptor (EGFR) are known to be the most crucial factors regulating epithelial cell restitution and proliferation [32]. The increased expression of EGFR is related to the performance of cell repair and migration in epidermal cells during inflammatory response [33]. 

The impacts of bioaerosols from food composting facilities on the inflammatory response and remodeling gene expression in airway epithelial cells are not well known. The current study determined the distribution of bacteria and fungi in the composting hall, maintenance area, and restaurant of a composting plant. The airborne endotoxin levels of the bioaerosols were determined in the indoor and outdoor composting hall to assess the correlation between endotoxin levels and particle matter. Additionally, this study investigated the change in the expression of remodeling genes (EGFR, TGF-β1, MMP-9, and p21^WAF1/CIP1^) in human airway epithelial cells exposed to the bioaersoals from a food composting plant.

## 2. Material and Methods

### 2.1. Field Sampling of Airborne Bioaerosols

**Figure 1:**
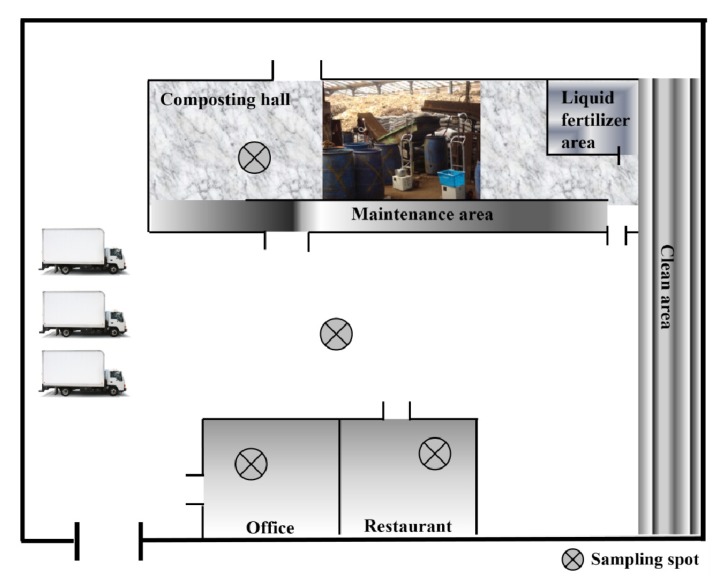
Layout of sampling pots of the food waste composting plant. The white color rea belongs an open access environment. The composting hall, maintenance, liquid fertilizer area, clean area, office and restaurant are closed building equipped with windows and ventilation system.

The layout of sampling locations in the composting plant is shown in Figure 1. The sampling of airborne microorganisms was carried out with a single stage ambient viable microbe sampler (TI-10-890, Tisch Environmental, Inc., Cleves, OH, USA) in the composting hall, the maintenance area and the restaurant of composting plant. The sampler with an aerodynamic cut-off size of 0.65 μm at a flow rate of 28.3 L/min was equipped with various agar media to isolate different groups of microorganisms. Meanwhile, for cytotoxicity assay in particulate matter of the composting hall, a filter cassette mounted with a polycarbonate membrane filter (0.4 μm pore size; SKC. Inc., Eighty Four, PA, USA) was used to collect particles at a flow rate of 4 L/min. The inlet of the one-stage viable sampler was located 1.5 m above the ground [34]. This study executed the 8-h bioaerosol sampling twelve times in the composting hall during the normal work period (8 a.m.–4 p.m.) from March to August.

In addition, a five-stage cascade impactor (Sioutas^TM^ Cascade Impactor, SKC Inc.) was used to collect particle samples for endotoxin assay. The sampler, equipped with polycarbonate membrane filters (0.4 μm) and Teflon membrane filters (2.0 μm), was operated at a flow rate of 9 L/min with an aerodynamic cut-off size of 2.5, 1.0, 0.5 and 0.25 μm. Particle sampling was carried out in the composting area 3 m from the product packing site and the inlet of impactor was 1.5 m above floor level. The weight of filter was measured before and after bioaerosol sampling. The sampling duration was 8 h per day during the normal work period indoors and outdoors in the composting hall. The sampling times were twice per month from March to August. 

### 2.2. Culture of Microorganisms from the Composting Facility

Microorganisms (microbes) are microscopic organisms that include bacteria, fungi, protozoa and virus, and can cause diseases or infections [35]. This study cultured *A*. *fumigatus*, bacteria and mould collected from the composting hall. Three types of culture based agars used in this study were Nutrient agar (NA, Difco^TM^, Becton Dickinson & Co., Sparks, MD, USA) for bacteria culture, Malt extract agar (MEA, Difco^TM^, Becton Dickinson & Co.) for *A*. *fumigatus* growth, and dichloran-glycerol agar base (DG18, Oxoid Ltd., Basingstoke, Hampshire, UK) for mould incubation. After sterilization in the autoclave progress, cycloheximide was added into NA culture base to inhibit mould growth, and chloramphenicol was used to inhibit bacterial contamination in MEA and DG18 culture based agar. 

### 2.3. Endotoxin Detection

Determinations of endotoxin in the filter of ambient field bioaerosol sampling were performed using kinetic *Limulus* amebocyte lysate (LAL) test kits (Associates of Cape Cod, Inc., East Falmouth, MA, USA). Sixty filters collected from different area around the composting sources were used for extraction and endotoxin assay. All materials used to detect endotoxin were pyrogen-free. To extract endotoxin, each filter was transferred into 5 mL LAL Reagent Water (LRW), followed by sonicating for 60 min at room temperature. The extracts were stored at −20 °C until determination of endotoxin concentrations. Lastly, the concentration of the endotoxin of composting plant was investigated using the standard Chromogenic-LAL-based assay (Lonza, Inc., Walkersville, MD, USA) as previously report [36] with minor modification. The five-point standard curve was generated ranging from 0.005 EU/mL to 50 EU/mL *Escherichia coli* O133:H10 reference endotoxin (*R^2^* > 0.998) with absorbance measured at 405 nm using a VERSAmax microplate reader (Molecular Devices, Sunnyvale, CA, USA). Levels of airborne endotoxins are presented as endotoxin units (EU) per milligram of particles (EU/mg) and as EU per cubic meter of sampled air (EU/m^3^).

### 2.4. Identification of A. fumigatus

A standard colony of *A*. *fumigatus* (ATCC 1028) were grown on the modified nutrient agar constituted with 31.5 g/L DG18 dichloran-glycerol agar base and 24 g/L potato dextrose broth (PDB). For obtaining the *A*. *fumigatus hyphaes*, a serial number of *A*. *fumigatus* conidia (10^2^, 10^3^, 10^4^, 10^5^, 10^6^) was cultured at a 26 °C vortex incubator for 7 days. Total RNA was isolated from the *hyphaes* using 1 mL RNA Trizol (Invitrogen, Carlsbad, CA, USA), and cDNA was synthesized from total RNA by a high-capacity cDNA reverse transcription kit (Applied Biosystems Inc., Foster City, CA, USA). In brief, 3 μg RNA was added 1.0 μL MultiScribe^TM^ reverse transcriptase (50 unit/μL), 2.0 μL 10X concentrated RT random primers, 0.8 μL 20X concentrated dNTP mix, 2.0 μL 10X RT buffer and RNase free water in a 0.2 mL PCR tube, and subsequently amplified by PCR with one cycle of 20 °C 10 min, 37 °C 120 min and 85 °C 5 s. 

### 2.5. Cell Culture of Human Airway Epithelial Cells

Human lung mucoepidermoid cells (NCI-H292; ATCC CRL-1848) were grown in a polystyrene tissue culture plate (BD Biosciences, San Jose, CA, USA) containing 90% RPMI 1640 (Invitrogen) culture medium with 1.5 g/L sodium carbonate (NaHCO_3_), 4.5 g/L glucose, 10 mL 1 M HEPES, 10 mL 100 mM sodium pyruvate (Sigma, St. Louis, MO, USA), 10% fetal bovine serum (FBS), and 1% 100X concentrated antibiotics (Invitrogen, Carlsbad, CA, USA). NCI-H292 cells were cultured in a 10-cm culture plate with 10 mL culture medium and maintained in the 37 °C, 5% CO_2_ cell incubator. 

### 2.6. Preparation of A. fumigatus/Field Bioaerosols Conditioned Medium

The co-culture of human lung mucoepidermoid cells (5 × 10^5^) with standard *A*. *fumigatus*/field bioaersol sample collected from composting hall were performed to mimic in exposure to inflammatory factors *in vivo*. The conditioned medium of the field sampled bioaerosol was prepared as the following procedure. The filter of field bioaerosol sampling was soaked into 3 mL RPMI culture medium with 15 min sonication after centrifuged, and the supernatant was stored at a −20 °C refrigerator to terminate biological activity. In the other hand, the conditioned medium of *A*. *fumigatus* (10^4^ conidia) was prepared by RPMI culture medium cultured with the standard colony of *A*. *fumigatus* for 24 h. After centrifuged, the *A*. *fumigatus* conditioned medium was stored at −20 °C.

### 2.7. Determination of Pro-Inflammatory Cytokine IL-6 Protein

The induction level of pro-inflammatory cytokine IL-6 was determined in NCI-H292 cells after treated with the conditioned medium of standard *A*. *fumigatus* / field bioaerosol samples for 24 h. After 24 h incubation in a 6 cm culture plate, the cultured medium was collected and centrifuged at 1500 rpm (10 min, 4 °C). The concentration of IL-6 secreted by H292 cells was determined using corresponding ELISA Ready-Set-Go kits (88-7066, eBioscience, San Diego, CA, USA). The optical density was detected at 450 nm within 30 min using a VERSAmax microplate reader (Molecular Devices, Sunnyvale, CA, USA). The level of IL-6 was converted from the absorbance value of a generating standard curve in the range of 2–200 pg/mL IL-6. 

### 2.8. Quantitative Real-Time PCR for Gene Expression

Total RNA was isolated from NCI-H292 cells in exposure to the 24 h condition medium of filed bioaerosols/*A*. *fumigates* using RNA Trizol (Invitrogen), and cDNA was synthesized from total RNA by a high-capacity cDNA reverse transcription kit (Applied Biosystems Inc., Foster city, CA, USA). In brief, 3 μg RNA was added 1.0 μL MultiScribe^TM^ reverse transcriptase (50 unit/μL), 2.0 μL 10X RT random primers, 0.8 μL 20X concentrated dNTP mix, 2.0 μL 10X concentrated RT buffer and RNase free water in a 0.2 mL PCR tube, and subsequently amplified by PCR with one cycle of 20 °C 10 min, 37 °C 120 min and 85 °C 5 s. The gene expression of TGF-β1, MMP-9, p21^WAF1/CIP1^, and EGFR were determined by quantitative real-time PCR. One hundred nanograms cDNA was analyzed using 2X power SYBR Green PCR master mix, forward and reverse primer, and RNase water-free water and determined by ABI 7300 sequence detection system (Applied Biosystem Inc.). The cycling condition of ABI prism 7300 real-time PCR system were 50 °C for 2 min, 95 °C for 10 min, and 95 °C for 15 sec, 55 °C for 30 s, 72 °C for 45 s for 40 cycles, with extension 95 °C for 15 s and 60 °C for 1 min. The relative level of mRNA expression was analyzed using comparative method by SDS 1.4 software normalized to the endogenous housekepping gene β-actin. The primers for *A*. *fumigatus* identification and mRNA expression of remodeling genes were shown in Table 1.

**Table 1:** Medium concentrations of airborne bacteria detected at various location of the composting plant.

Sampling Site	Bacteria		Fungi	
	Mesophiles (CFU/m^3^) (n)	Thermophiles (CFU/m^3^) (n)	Mesophiles (CFU/m^3^) (n)	Thermophiles (CFU/m^3^) (n)
Composting	4.0 × 10^4^ * (64) (1.3 × 10^3^–3.7 × 10^5^)	1.7 × 10^4^ (37) (1.0 × 10^3^–1.9 × 10^5^)	5.4 × 10^3^ (16) (9.2 × 10^2^–1.4 × 10^4^)	3.4 × 10^3^ (8) (3.2 × 10^2^–8.8 × 10^3^)
Maintenance	1.4 × 10^4^ * (24) (2.3 × 10^3^–4.0 × 10^4^)	6.3 × 10^3^ (16) (1.5 × 10^2^–1.4 × 10^4^)	4.1 × 10^3^ (8) (2.3 × 10^3^–8.7 × 10^3^)	3.7 × 10^3^ (4) (1.3 × 10^3^–8.5 × 10^3^)
Restaurant	1.7 × 10^3^ * (11) (4.3 × 10^2^–4.3 × 10^3^)	2.0 × 10^2^ (9) (2.6 × 10^1^–4.4 × 10^2^)	1.0 × 10^3^ * (6) (6.8 × 10^1^–2.4 × 10^3^)	1.2 × 10^2^ (4) (8.0 × 10^1^–2.0 × 10^2^)

Notes: CFU: colony-forming unit; * Asterisk indicates significant differences between mesophilic and thermophilic bacteria/fungi respectively in the composting hall, maintenance area and restaurant of the composting plant (*p* < 0.05).

### 2.9. Statistical Analysis

Data analysis was conducted by Statiscal Package for the Social Science (SPSS), version 12.0 (SPSS Inc., Chicago, IL, USA). Data are reported as means ± SD. Mean concentrations of field aerosol particle in indoor and outdoor environment of the composting hall were compared using Mann-Whitney U test. The standard curves for quantifying *A*. *fumigatus* and IL-6 ELISA were individually plotted with Microsoft Excel Statistic Suite. Statistically significant differences of IL-6 and gene expression of exposure groups to *A*. *fumigatus* and field bioaerosols sample were analyzed using Student’s *t* test. All statistical significances were determined at two-tailed *p* value < 0.05. 

## 3. Results and Discussion

### 3.1. Quantification of A. fumigatus

Culture-dependent methods are known to be biased because bacteria can only be cultured when their metabolic and physiological requirements are reproduced *in vitro* [37]. Epifluorescence microscopy uses fluorochrome stains to count total microbes, but cannot to distinguish between different species of microorganisms [38]. Both viable and nonviable bioaerosols can cause infections. Thus, quantitative real-time PCR is a reliable detection method for total airborne bacteria assessment. Rinsoz *et al*. [38] found that the estimated number of *Staphylococcus spp*., determined using real-time PCR, had a 23-fold increase compared to the number estimated by direct impact on selective nutrient media. In this study, a standard curve of Ct value *versus* the number of conidia was generated using real-time PCR to identify and quantify the gene expression level of a standard colony of *A*. *fumigatus*. Ct values between 25.9 and 33.5 corresponded to 10^6^ to 10^2^ *A*. *fumigatus* conidia. The Ct values of *A*. *fumigatus* in field bioaerosol samples from the composting hall were in the range of 34.5 to 36.1, being lower than that of 10^2^ *A*. *fumigatus* conidia, according to the standard curve. However, because there is neither a universally recognized technique nor standardization of monitoring equipment for bioaerosol monitoring, the reported standard number of *A*. *fumigatus* in the workplace has been inconsistent.

This study used a standard strain of *A*. *fumigatus* to quantify the amounts of *A*. *fumigaus* present in the composting hall. The results of real-time PCR indicated that the amount of *A*. *fumigatus* in the composting hall was lower than that of 10^2^ conidia. Vanhee *et al*. [39] determined the number of *A*. *fumigatus* using solid-phase cytometry, and reported a low 5 ± 3 to high 57 ± 38 number of conidia in environmental air samples. Environmental *A*. *fumigatus* dispersion has been studied in various working areas such as a composting facility [40], hospital [41] and sewage sludge disposal area [42]. Typically, concentrations of up to 10^5^ CFU/m^3^ of *A*. *fumigatus* are not considered a risk for healthy individuals [43]. However, the 90% mortality rate they observed in immunocompromised transplant recipients was due to severe infections such as aspergilloma, chronic necrotizing aspergillosis, IPA, and ABPA [44]. Therefore, scrupulous monitoring of *A*. *fumigatus* is required specifically for patients with immunodeficiency (e.g., newborns and transplantation patients).

**Figure 2:**
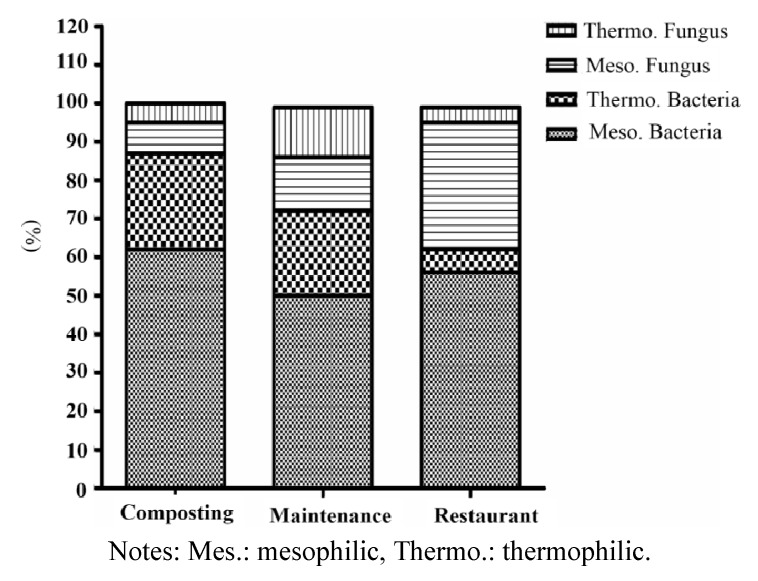
Distribution of microorganisms in the composting hall, maintenance and restaurant.

Notes: Mes.: mesophilic, Thermo.: thermophilic.

### 3.2. Microbe Concentrations in Different Areas of the Composting Hall

Compost workers are at risk of suffering respiratory tract diseases [45]. Bioaerosols are airborne particles consisting of living or dead organisms, as well as metabolites, toxins, and fragments of microorganisms [46]. The term bioaerosol includes all airborne microorganisms regardless of viability or non-viability, solubility, or insolubility [47]. The airborne particles of bioaerosols consist of various microorganisms (bacteria, virus, and molds), metabolites, toxins or fragments of microorganisms. In this study, microorganism concentrations were determined for field samples from the composting hall, maintenance area, and restaurant of the composting plant. The layout of the sampling sites in the food waste composting facility is shown in Figure 1. Mesophilic bacteria predominated, representing 62% of all microorganisms in the composting hall, and 50% and 56% of all microorganisms in the maintenance and restaurant area of the composting plant, respectively (Figure 2). The levels of mesophilic bacteria were significantly higher than those of thermophilic bacteria in all the tested areas (*p* < 0.05). The proportion of microbes distributed in the maintenance as well as in the composting hall. In the restaurant, mesophilic fungi (33%) were the second most abundant microorganisms after mesophilic bacteria (56%). The concentrations (colony-forming units per cubic meter; CFU/m^3^) of airborne bacteria detected at various location of the composting plant are presented in Table 1. A temperature range of 25 °C (77 °F) to 45 °C (113 °F) is the most suitable for the reproduction and growth of mesophilic microbes. Critically, a temperature range between 36 °C (90 °F) and 62 °C (140 °F) is produced during the compost pile process for rapid composting [48,49]. In addition, a large number of mesophilic bacteria (53%) and mesophilic fungi (33%) were detected in the restaurant of the composting plant, which agrees with a previous study [26].

### 3.3. Airborne Particle Concentration and Endotoxin Levels in the Composting Plant

Endotoxins generated by bacteria in bioaerosols are the most common triggers for allergic reactions that exist within living environments, and are a potential respiratory hazard to individuals. Based on the aerosol-dynamic processes, both coarse and fine particles from environmental aerosols are inhalable and can deposit in the respiratory system [50]. Fine particles deposit easily in the respiratory bronchioles and alveoli and in the tracheobronchial region. The respiratory deposition of particles is generally related to the particle size. Air particle in the range of PM 0.5–2.5 μm can deposit in distal airways and alveoli, PM 2.5–10 μm deposit in larynx and conducting airways, and PM > 10 μm in nasopharynx deposition [50]. Thus, broad spectrum monitoring of ambient aerosols in the environment is necessary to prevent potential respiratory system irritation. This study determined the average concentrations of airborne coarse (2.5–10 μm) and fine airborne particles (0.25–2.5 μm) in the indoor composting hall were 36.0 μg/m^3^ and 96.9 μg/m^3^, respectively, whereas in the outdoor composting hall they were 19.0 μg/m^3^ and 34.7 μg/m^3^, respectively (Table 2). 

**Table 2:** Average concentration of endotoxin in airborne sampled coarse and fine airborne particles.

Sampling Site	Particle Meter (PM)	Airborne Particle Concentration (μg/m^3^)	EU/m^3^	EU/mg	EU/mg (%)
Indoor	Coarse particle (2.5~10 μm)	36.0	32.9 ± 47.1	913.9	68.7
	Fine particle (0.25~2.5 μm)	96.9	40.4 ± 2.1	416.9	31.3
Outdoor	Coarse particle (2.5~10 μm)	19.0	12.7 ± 15.1	668.4	59.8
	Fine particle (0.25~2.5 μm)	34.7	15.6 ± 5.5	449.6	40.2

Notes: EU: endotoxin unit; endotoxin (EU/mg) = endotoxin (EU/m3)/particle concentration (μg/m3); EU/mgcoarse (%) = [EU/mgcoarse/(ER/mgcoarse + EU/mgfine)] × 100; EU/mgfine (%) = [EU/mgfine/(ER/mgcoarse + EU/mgfine)] × 100.

By contrast, the endotoxin content (endotoxin units/mg, EU/mg) of the coarse particles in the indoor composting hall was higher (913.9 EU/mg) than that of the fine mass fraction (416.9 EU/mg). Moreover, the coarse particles in the outdoor composting hall exhibited a higher endotoxin level (668.4 EU/mg) than did the fine particles (449.6 EU/mg). A 2-year study of the correlation of ambient inhalable bioaerosols and particulate matter was conducted in Cincinnati (OH, USA) and reported higher airborne concentrations of biological and non-biological pollutants in PM10 (6.7–65.38 μg/m^3^) than those in PM2.5 (5.04–45.02 μg/m^3^) [51]. In Germany, sampling of the endotoxin content in two cities found that the coarse (PM2.5–10) particle fraction exhibited endotoxin levels 10-times higher than those of the fine (PM2.5) particle fraction [52]. Size distribution analysis of airborne endotoxins in biofuel plants showed a high concentration for the thoracic fraction (PM10) [53]. In a cohort study conducted in Fresno (CA, USA) [54] observed that the highest daily endotoxin content of coarse particulate matter presented temporal and spatial variations. In summary, the coarse airborne particles had a higher endotoxin level than did the fine airborne particles. 

### 3.4. IL-6 Secretion in NCI-H292 Cells Exposed to a Conditioned Medium of Field Bioaerosol Samples

Bioaerosols and *A*. *fumigatus* are known to stimulate human airway epithelial cells to release inflammatory hormones [2]. Respiratory epithelium cells have a defensive role against external pathogens. IL-6 is known to be a reactive pro-inflammatory cytokine that responds to microorganisms and invasive toxins. Activation of IL-6 induces a transcriptional response, and is a potent inducer of the acute phase response (e.g., inflammation and acute respiratory distress). Indeed, after intranasal exposure to lipopolysaccharides (LPS), there was a significant increase in all measured cytokines (TGF-α, IL-1β, IL-6, and IL-8) in the blood of five refuse workers with occupational airway symptoms, compared with five healthy refuse workers [55]. In addition, IL-6 cytokine is a pro-inflammatory cytokine known to reduce apoptosis by suppressing TGF-β1 performance, and it also causes the p21^WAF1/CIP1^ promoter to express p21^waf^ to protect cells from apoptosis [56]. 

**Figure 3:**
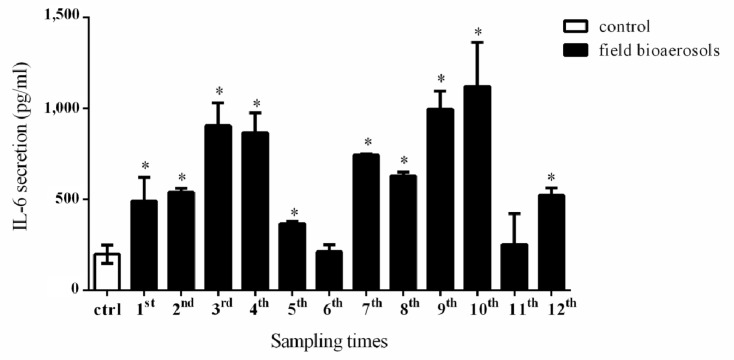
IL-6 secretion of NCI-H292 cells cultured with field bioaerosol samples. Confluent NCI-H292 cells (5 × 105) were individually cultured with the conditioned medium of field bioaerosol samples for 24 h. Asterisk indicated significant at *p* < 0.05 compared with the control group.

Furthermore, the activation of IL-6 is known to induce a transcriptional response, and it is a potent inducer of the acute phase response (e.g., inflammation and acute respiratory distress). IL-6 triggers the expression of TGF-β to promote differentiation of naive T lymphocytes into pro-inflammatory IL-17, to further defend against an external infective agent [57]. In this study, the results of MTT cell viability assay indicated that the conditioned medium of field bioaerosols or 10^4^ *A. fumigatus* conidia had no obviously cell death to NCI-H292 cells, up to 24 h co-cultured duration. In addition, we used the same amount 3 ug RNA sample of airway cells after field bioaerosol exposure to perform the gene expression quantization in PCR reaction. Exposure to field bioaerosol samples induced a higher secretion of IL-6 in the co-culture with NCI-H292 cells (306-1477 pg/mL) than control group (188 pg/mL). Figure 3 shows that NCI-H292 cells secreted IL-6 after 24 h exposure to bioaerosols conditioned medium.

### 3.5. Exposure to Bioaerosol or A. fumigatus Mediated Gene Expression

Bioaerosols could be liquid or solid particles suspended in a gaseous medium, having size ranges from 0.001 to 100 μm [58]. Because of their complex constituents, bioaerosols release allergenic factors that induce health problems including infectious diseases, respiratory diseases and cancer [59]. Various components (e.g., ammonia, microbes, endotoxins and fungi) released by daily composting could threaten the health of workers in a composting facility. Bunger *et al*. [8] conducted a 5-year follow-up study and observed that workers exposed to organic dust from composting plants had adverse acute and chronic respiratory effects such as mucosal membrane irritation (MMI), chronic bronchitis, and an accelerated decline of forced vital capacity (FCV%). However, it has been unclear how the bioaerosol from a food composting hall influences the expression of remodeling genes in airway epithelial cells. This study explored the expression change of remodeling genes in human airway epithelial cells exposed to the conditioned medium of field bioaerosol samples from the composting hall of a food composting plant and *A*. *fumigatus* standard strain (10^4^ condia), and compared the obtained data with that of a control group. 

**Figure 4:**
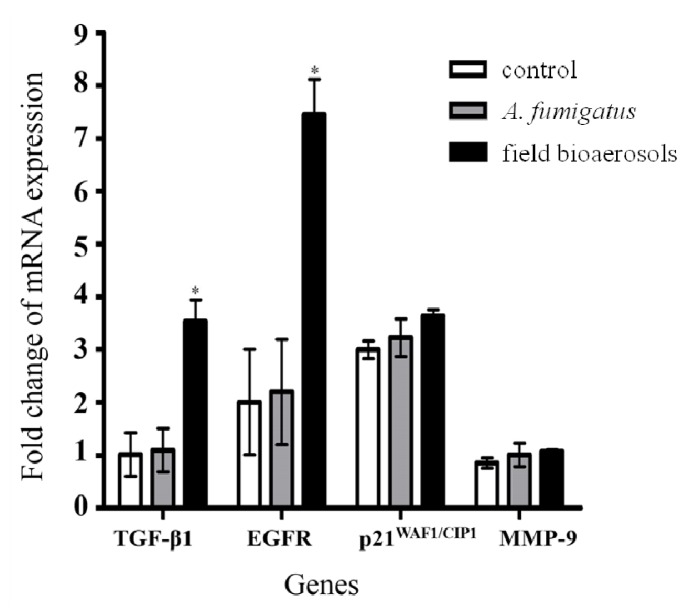
Quantitative expression of remodel genes in NCI-H292 cells treated with control group, standard A. fumigatus or field bioaerosol samples for 24 h. The bars were represented the mean fold change of mRNA expression between control group and treatment group at least three independent experiments. Asterisk indicated significant at *p* < 0.05 compared with the control group.

The mRNA expression of TGF-β1, EGFR, p21^WAF1/CIP1^, and MMP-9 in H292 cells treated with the conditioned medium of field bioaerosol samples or standard *A*. *fumigatus* was determined using real-time PCR. NCI-H292 cells exposed to field bioaerosol sample for 24 h significantly induced the mRNA expression of TGF-β1, EGFR and p21^WAF1/CIP1^ (fold change 3.7 ± 0.42, 7.5 ± 0.45 and 3.6 ± 0.23; Figure 4), but it had no significant effect on MMP-9 expression. NCI-H292 cells treated with standard *A*. *fumigatus* (10^4^ conidia) resulted in no augmented expression of TGF-β1, EGFR, MMP-9, or p21^WAF1/CIP1^. TGF-β and EGFR are involved in the most notable signaling pathway for epithelial restitution. The activated TGF-β1 and EGFR expression initiates cellular interactions such as cell differentiation, proliferation and apoptosis, which result in the transformation and remodeling of epithelial cells [56]. Upregulation of TGF-β1 activation may result in airway epithelial cell growth and proliferation, anti-apoptosis activity, and cell survival by phosphorylation of downstream EGFR. EGFR activation involves airway epithelial repair through cell migration. A culture of asthmatic bronchial epithelium treated using TGF-β resulted in p21 becoming nuclear, suggesting interactions with the replicative machinery and implying a link to cell survival [60]. After a 24 h, neither NCI-H292 cells exposure to the field bioaerosols nor the *A*. *fumigatus* exerted an influence on the MMP-9 gene expression. This might be due to the upregulated p21 involved cells toward cell cycle arrest further to inhibit the protease bioactivity of MMP-9. This suggested that p21 ^WAF1/CIP1^ upregulation interrupts programmed cell death. The activation of TGF-β1 induces the p21^WAF1/CIP1^ pathway to inhibit cyclin D-dependent kinase 4, which is required for cell cycle arrest [61]. Moreover, this result was correlated with the further phosphorylation of EGFR induced by TGF-β1, which activates the STAT-3 signaling pathway, and consequently reduces the MMP-9 expression [62]. In this study, NCI-H292 cells exposed to *A*. *fumigatus* did not exhibit obvious expression differences in the regulation of remodeling genes, suggesting that the amounts of field bioaerosols or *A*. *fumigatus* in the composting hall were not sufficient to damage the NCI-H292 cells immediately, resulting in the unchanged expression of MMP-9. However, respiratory epithelial remodeling is a dynamic process that occurs in combination with a variety of peptides, enzymes, and proteases, where small changes in the levels of intermediate cellular factors may alter the equilibrium of bioactive proteins in the submucosal layer. 

## 4. Conclusions

**Figure 5:**
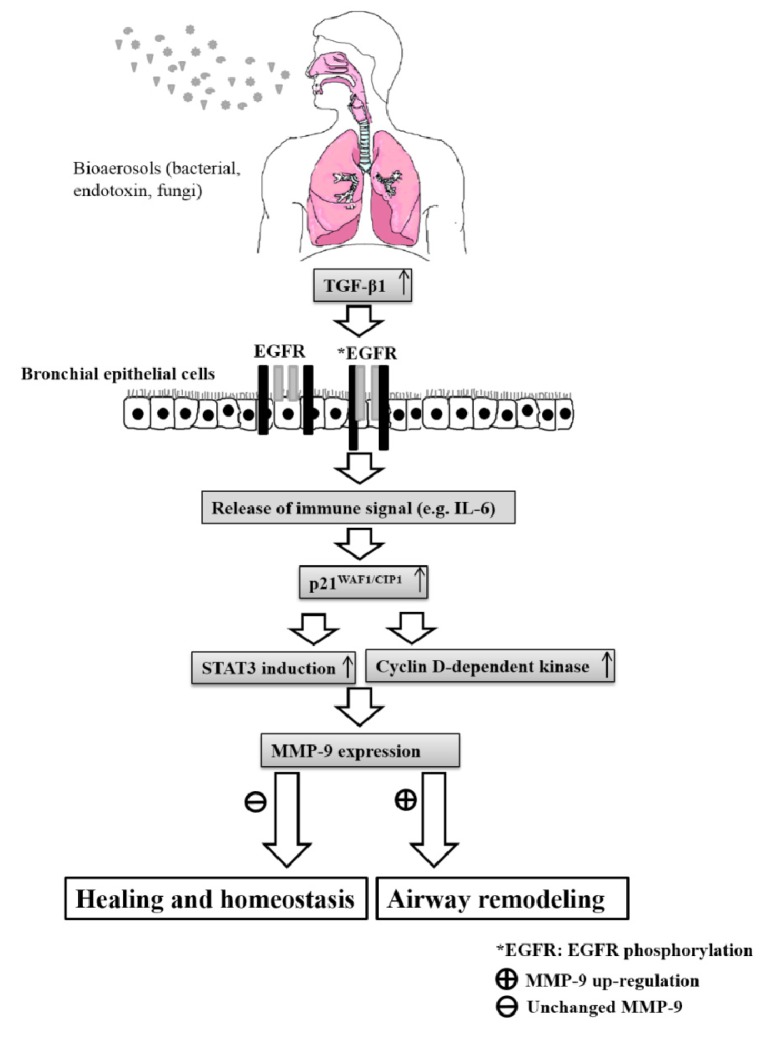
A possible mechanism of airway remodeling genes in response to bioaersol exposure in the composting hall. The atopic inflammation is triggered when innate immune cells of bronchial epithelial cells are exposed to bioaerosol allergens. The activation of IL-6 further recruits the inflammatory cells, and consequently programs epithelial cells to healing or remodeling. In addition, TGF-β1 activates EGFR phosphorylation and increases p21^WAF1/CIP1^ expression that implicates airway remodeling due to irregular cell proliferation and prolonged repair duration. The unchanged MMP-9 expression might be compensated for the over-expression of STAT-3 and cyclin D-dependent kinase. Consequently, both the atopy reaction and ligand induced activation of EGFR signaling cascade are to tend toward to guide cells healing or remodeling.

This study observed that the expression of remodeling genes, including TGF-β, p21^WAF1/CIP1^ and EGFR was upregulated after exposure to field-sampled bioaerosols. The possible mechanism of airway remodeling response of genes to bioaerosols from the composting hall is illustrated in Figure 5. Importantly, MMP-9 regulation might be a crucial factor for programmed epithelial cell healing or remodeling, which relies on the upstream signaling pathway of STAT-3 and cyclin D-dependent kinase. Additionally, this study investigated the distribution of microorganisms in the composting hall, maintenance area, and restaurant of a composting plant. The results showed that both the composting hall and maintenance area contained an abundance of mesophilic bacteria (62% and 50%, respectively). The current study observed the endotoxin concentrations of airborne coarse particles (2.5–10 μm) presented per mg were higher than those of fine particles (0.25–2.5 μm), irrespective of the indoor or outdoor location of the composting hall. Inside the composting hall, the levels of airborne endotoxins were higher in particulate matters between 2.5 and 10 μm (coarse) than they were in fine particles. By contrast, NCI-H292 cells treated with standard *A*. *fumigatus* showed no prominent change of gene expression. *A*. *fumigatus* is ubiquitous in both indoor and outdoor environments, and is often measured as an assessment criterion for the health condition of workers and the surrounding neighborhood. 

According to our results, the levels of *A*. *fumigatus* were not an effective indicator for health monitoring because of its relatively low abundance, so it would underestimate the impact on the health of workers. Therefore, this study suggests that using only one species for environmental monitoring is inadequate. Regarding the low amount of *A*. *fumigatus* in the composting hall analyzed and the remodeled gene profile after exposure to the sampled bioaerosols, this study proved that endotoxins are one of the major hazard materials during airway remodeling and repair processes. Furthermore, the endotoxin concentrations in the indoor and outdoor composting hall reported in this study were lower than the health-based guidance limit of 50 EU/m^3^ recommended for occupational settings in the Netherlands [63]. The conglomerate nature of pathogens influences health conditions extensively and comprehensively. Additional, studies on pathogen diversity in bioaerosols and their effect on the airway symptoms of workers in food waste composting site are necessary.
